# Composition and diversity analysis of the TCR CDR3 repertoire in patients with idiopathic orbital inflammation using high-throughput sequencing

**DOI:** 10.1186/s12886-023-03248-x

**Published:** 2023-12-04

**Authors:** Yenan Fang, Bingyan Shen, Qin Dai, Qiqi Xie, Xinyu Li, Wencan Wu, Min Wang

**Affiliations:** 1 National Clinical Research Center for Ocular Diseases, Eye Hospital, Wenzhou Medical University, Wenzhou, 325027 China; 2 Department of Ophthalmology, Children’s Hospital of Fudan University, National Children’s Medical Center, No. 399 Wanyuan Road, Shanghai, 201102 China; 3 State Key Laboratory of Ophthalmology, Optometry and Visual Science, Eye Hospital, Wenzhou Medical University, Wenzhou, 325027 China

**Keywords:** Idiopathic orbital inflammation, T-cell receptor, High-throughput sequencing, Glucocorticoid sensitivity

## Abstract

**Background:**

Idiopathic orbital inflammation (IOI) is a nonspecific orbital inflammatory disease with the third highest prevalence among orbital diseases, and its pathogenesis is associated with T-cell-mediated immune responses. This study aimed to investigate the differences in T-cell receptor (TCR) expression between IOI patients and healthy subjects by high-throughput sequencing and to characterize TCR expression in patients with IOI and with respect to glucocorticoid response.

**Methods:**

A total of 19 subjects were enrolled in this study and were divided into the idiopathic orbital inflammation group (IOI group, n = 13) and the healthy control group (HC group, n = 6), and within the IOI group were further divided into the glucocorticoid therapy sensitive group (IOI(EF) group, n = 6) and the glucocorticoid therapy ineffective group (IOI(IN) group, n = 7) based on the degree of effectiveness to glucocorticoid therapy. High-throughput TCR sequencing was performed on peripheral blood mononuclear cells of IOI patients and healthy control individuals using 5’ RACE technology combined with Unique Identifier (UID) digital tag correction technology. The TCR CDR3 region diversity, sharing patterns, and differential sequences between the IOI and HC groups, and between the IOI(EF) and IOI(IN) groups were analyzed.

**Results:**

It was found that the diversity of TCR CDR3 in the IOI group was significantly lower than that in the HC group, and the frequency of V gene use was significantly different between groups. The diversity of TCR CDR3 in patients in the IOI(EF) group was significantly lower than that in patients in the IOI(IN) group, and the frequency of V and J gene use was significantly different between the IOI(EF) group and the IOI(IN) group. Additionally, we found 133 nucleotide sequences shared in all IOI samples and screened two sequences with higher expression from them.

**Conclusions:**

Our results suggested that abnormal clonal expansion of specific T-cells exists in IOI patients and that TCR diversity may had an impact on the prognosis of glucocorticoid-treated IOI. This study may contribute to a better understanding of the immune status of IOI and provide new insights for T-cell -associated IOI pathogenesis, diagnosis and treatment prediction.

**Supplementary Information:**

The online version contains supplementary material available at 10.1186/s12886-023-03248-x.

## Introduction

Idiopathic orbital inflammation (IOI), also known as orbital inflammatory pseudotumor (OIP), accounts for approximately 8-10% of all orbital occupying lesions and is a benign, nonspecific orbital disease [1]. It can cause orbital pain, swelling, diplopia, proptosis, limited eye movement, and loss of vision [2]. Treatment options for IOI include glucocorticoid therapy, radiation therapy, and immunosuppressive therapy, among these interventions, glucocorticoid therapy is the most commonly used. The efficiency of glucocorticoid therapy for IOI has been reported to be 63-87% [3–5]. This indicates that different IOI patients have different sensitivities to glucocorticoids, but the specific mechanism is not clear. Since long-term glucocorticoid use has many side effects (e.g., induced hypertension, induced glaucoma, and aggravated gastrointestinal ulcers), there is an urgent need to find biomarkers that correlate with patients’ glucocorticoid treatment responses to select more appropriate treatment regimens and reduce adverse effects in patients.

Numerous previous studies have shown that the pathogenesis of IOI is closely related to abnormal immune responses [6–8], especially T cell-mediated immune responses [9–11]. T cells play a key role in adaptive immunity by recognizing antigens through the membrane protein T-cell receptor (TCR) [12]. In humans, the αβ T cell population is the majority of the total T cell population [13], and the β chain has a higher rearrangement potential compared with the α chain [14], so the vast majority of studies have taken the TCR β chain encoded by the TRB gene as the main target region. For the compositional structure, TCR consists of variable (V), diverse (D), joining (J), and constant (C) regions [15]. The TCR has three complementarity-determining regions (CDRs), of which CDR3 is in direct contact with the antigenic peptide bound by the major histocompatibility complex molecules and plays a major role in interaction with peptide-MHC complexes [16–18]. CDR3 is the most variable region, which largely determines the diversity of TCR [19]. Complex recombination of VDJC gene fragments, random insertion or deletion of nucleotides in the V-D and D-J linkage regions, and mutations in somatic cells all contribute to the diversity of the TCR. A diverse TCR repertoire is the basis of the adaptive immune system and reflects, to some extent, the adaptive immune state. In some disease states, targeted and selective rearrangements of TCR genes occur, and clonal proliferation of certain TCR-specific T cells may occur.

At present, most studies related to the pathogenesis of IOI are based on changes in the number of T-cell -associated subpopulations and T-cell activation and proliferation-related cytokines [20]. For example, patients with IOI have been found to have increased regulatory T cells (Tregs) in circulating blood and affected orbital tissues compared to healthy individuals [9, 10].Tregs with proinflammatory and profibrotic polarization were increased in the IOI, presumably playing a role in this disease. To further study the role of T cells in the pathogenesis of IOI, we need a deeper understanding of the sequence information and diversity changes of TCR in IOI patients.

The advent of high-throughput sequencing (HTS) technology has enabled scientists to study gene pools at an unprecedented level. As the sensitivity and convenience of TCR detection improve, the technique allows for more precise assessments of TCR CDR3 diversity and clonal composition, including library size, similarity between fragments, use of VDJ fragments, nucleotide insertions and deletions, CDR3 length, and amino acid distribution at the level of the CDR3 sequences [21], leading to a comprehensive understanding of the pattern of the human TCR CDR3 repertoire. In addition, through TCR sequencing, it is possible to identify clonal amplification of specific sequences in IOI, which is expected to enable specific diagnosis, targeted therapy, and therapeutic monitoring [22].

In this study, we analyzed the unique characteristics of the TCR in IOI patients by high-throughput sequencing of the TCR and compared the differences in TCR expression between glucocorticoid-sensitive and glucocorticoid-ineffective patients to provide a further basis for elucidating the pathogenesis of the disease, finding new targets for its clinical immune intervention, and predicting the efficacy of glucocorticoids in advance.

## Materials and methods

### Basic information of subjects

This research project was approved by the ethics committee of the Wenzhou Medical University Affiliated Eye Hospital(2022-018-K-15-01). Written informed consent was obtained from all individuals participating in the study. Peripheral blood samples were obtained from 13 patients with IOI (IOI group) and 6 healthy control individuals (HC group). The diagnostic criteria for IOI were that the clinical presentation, signs, and imaging features were consistent with the 2017 JAMA consensus on the diagnosis of IOI, and all were diagnosed with IOI by the same experienced ophthalmologist. Patients with IOI who had not received any systemic therapy (including immunosuppressants and glucocorticoids) for at least 4 weeks prior to the trial were included in the IOI group [23]. Patients with other immune-related diseases or with contraindications to glucocorticoids were excluded from the study. The HC group did not develop IOI or other autoimmune diseases.

Disease activity scores were determined according to the modified Werner classification before and after glucocorticoid therapy at 1 week, 1 month, 3 months, 6 months and 12 months. Improvement was defined as a reduction in disease total score of 2 points or more, or a reduction in the total score of 3 points or less [24]. When both eyes were affected at the same time, the score was based on the affected eye with severe disease. If the patients met the criteria for improvement, they were considered effective with glucocorticoid treatment and were included in the IOI(EF) group (6 subjects), otherwise they were included in the IOI(IN) group (7 subjects).

We collected basic patient information, compiled the detailed medical histories of all patients, performed a comprehensive ophthalmologic examination, and collected the patients’ spherical equivalent (SE), best corrected visual acuity (BCVA), intraocular pressure (IOP), ocular prominence, and the results of computed tomography (CT) (Siemens Dual Source CT, Germany) and/or magnetic resonance imaging magnetic resonance imaging (MRI) (Siemens Magnetom Avanto 1.5T, Germany).

### Glucocorticoid treatment regimen for patients with IOI

The patients were given intravenous methylprednisolone sodium succinate (Miloxone) 500 mg/d for 7 days. Additionally, the patients were given potassium supplements, stomach protection, and calcium supplement treatment. After 7 days of high-dose glucocorticoid therapy, prednisone acetate tablets (60 mg) were given orally once a day, and 2 tablets were reduced every 5 days. The therapeutic effect of patients was evaluated again at least 2 months after therapy.

### RNA extraction and CDR3 region amplification

Patients’ peripheral blood samples (5 ml) were collected before treatment and at the last follow-up after treatment, as well as peripheral blood samples (5 ml) from healthy individuals, which were preserved in EDTA anticoagulation tubes and stored at a -80 °C refrigerator. Blood samples in the frozen state were milled in a liquid nitrogen environment and RNA was extracted using TRIzol™ LS (10,296,010, Thermo Fisher). Approximately 2 µg of RNA per sample was taken for TCR sequencing library preparation.

Then, the library was constructed with the KC-Digital™ Stranded TCR-seq Library Prep Kit (DT0811-02, SeqHealth). The kit first added a unique UID sequence consisting of eight random bases to each RNA fragment in the CDR3 region by Unique Identifier (UID) tagging technology, and then builds the library by amplification of the target region with PCR. Then the single-stranded cDNA was obtained by 5 ‘RACE reverse transcription with random primers, and the target region was amplified by PCR to construct the library.

### High-throughput sequencing and data analysis

Sequencing was performed on the Illumina Novaseq 6000 sequencing platform. Raw image data obtained by high-throughput sequencing was converted into raw sequence data in FASTQ format by Base Calling software.

The raw sequencing data was first filtered by SOAPnuke(version 1.6.0) to obtain high-quality sequencing data (clean data). Data quality control (QC) was completed using Fastp (version 0.23.0) software [25], followed by error correction and removal of duplicate reads using kcUID software, comparative analysis of reads using MiXCR (3.0.13) software, and finally comparison with V, D, and J gene fragments in the IMGT database [26, 27]. The genetic information, rearrangements, and CDR3 sequences of the reads could be obtained by comparison.

After the comparison, the TCR rearrangement function of each sample was classified according to the functional annotation of the V/J gene in IMGT, the length of the CDR3 region, and the CDR3 coding product. Then the expression level of each clonotype was calculated. The clonotype frequencies and frequency distributions of DNA sequences, amino acid sequences, and V-J combinations in the CDR3 region were analyzed. Shannon entropy and D50, indicators for calculating the diversity of the TCR composition of the samples, were used [28]. The Shannon entropy was calculated by the following formula: $\text{H}=-\sum _{i=1}^{{\rm s}}\left({\text{p}}_{{\rm i}} \text{lnp}_{{\rm i}}\right)$ where s denotes the actual number of observed TCR recombination sequences, and ${\text{p}}_{{\rm i}}$ denotes the proportion of the ith TCR rearranged sequence among all TCRs. D50 is the amount of TCR rearranged sequences in the sample when the proportion of TCR rearranged sequences in the sample reaches half of the sample. The larger the Shannon entropy and D50 value, the higher the TCR diversity of the sample.

Independent samples t test and Welch’s t test were used to assess differences between groups. Analyses were performed using GraphPad Prism 8.0.2. P < 0.05 was considered statistically significant.

## Results

### Basic information of the subjects

A total of 13 subjects in the IOI group and 6 subjects in the HC group were included in this study. Before treatment, blood samples were collected from all subjects in the IOI and HC group. Unfortunately, after the treatment of glucocorticoids, we only received blood samples of 6 patients in IOI group.

**Fig. 1:**
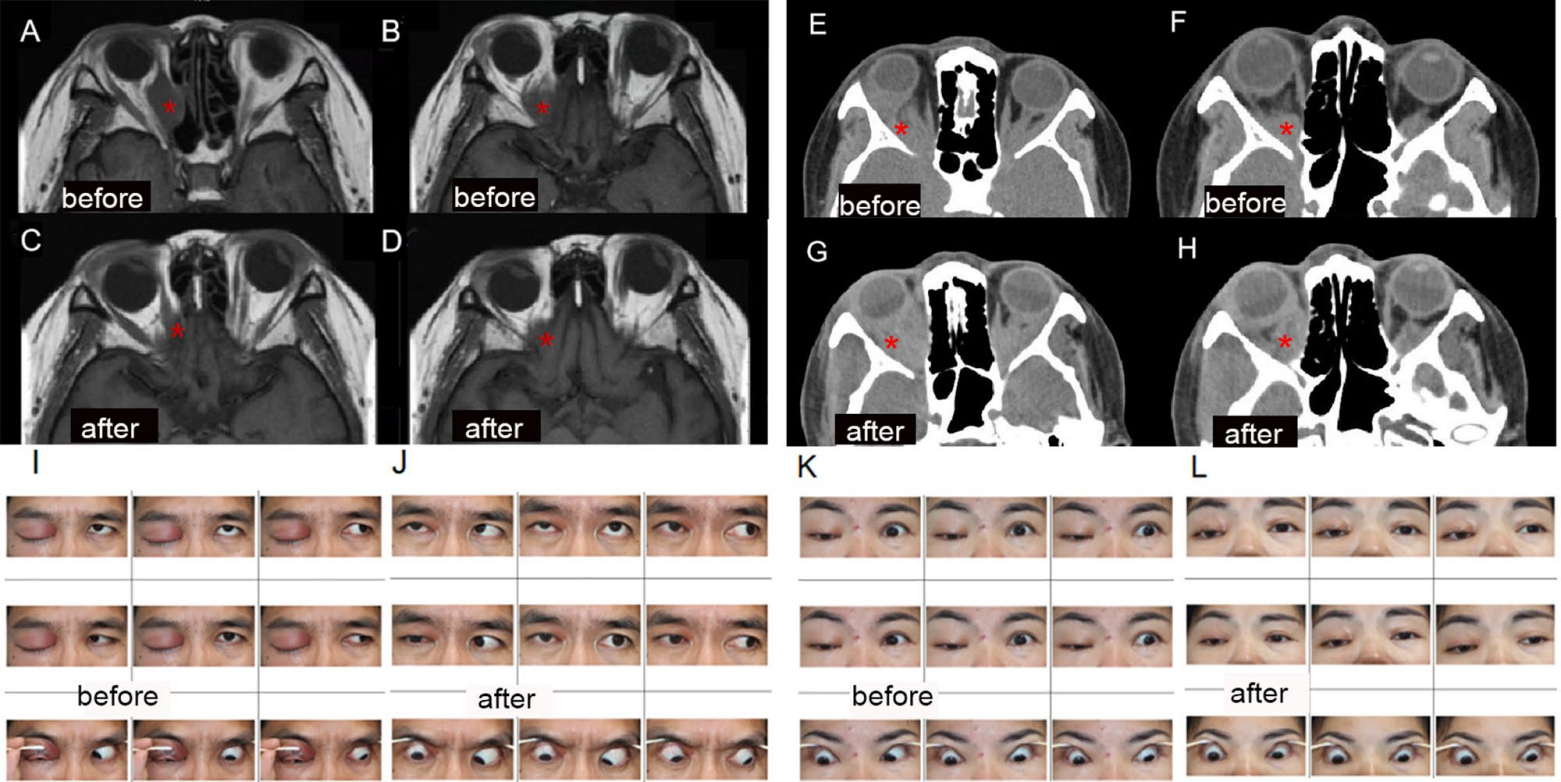
Comparison of clinical examinations in the IOI(EF) group and the IOI(IN) group. (**A. B**): MRI of IOI03 in the IOI(EF) group showed swelling of the right eye extraocular muscle before treatment. (**C. D**): After treatment, MRI showed obvious improvement in the above symptoms of IOI03. (**E. F**): CT of IOI07 in the IOI(IN) group showed swelling of the extraocular muscle of the right eye. (**G. H**): CT of IOI07 after treatment showed no improvement of the above symptoms. (**I**): IOI01 in IOI(EF) group showed obvious swelling of the upper eyelid of the right eye before treatment. (**J**): After treatment, the above symptoms of IOI01 were significantly improved. (**K**): IOI07 in the IOI(IN) group showed ptosis of the upper eyelid and swelling of right eye before treatment. (**L**): The above symptoms of IOI07 did not improve after treatment

A comparison of the basic information of the IOI and HC groups is shown in Table 1, and the differences in BCVA and D50 between the two groups were statistically significant. The mean follow-up and scoring time of the 13 patients in the IOI group was 6.69 ± 4.03 months. The patients in the IOI group were scored for disease activity before and after treatment according to the modified Werner grading method [24] (Supplemental Table S1). Figure 1 shows the comparison of clinical examinations in the IOI(EF) group and the IOI(IN) group.

**Table 1:** Basic information of the subjects

Characteristics	HC group	IOI group	IOI(EF) group	IOI(IN) group	*P* Value_*1*_	*P* Value_*2*_	*P* Value_*3*_	*P* Value_*4*_
N	6	13	6	7	-	-	-	-
Age, year	41.83 ± 12.64	43.31 ± 13.91	38.17 ± 16.71	47.71 ± 8.84	0.837	0.704	0.386	0.253
Gender, F:M	4: 2	9: 4	3:3	6:1	0.911	0.558	0.416	0.164
SE, D	-0.44 ± 1.78	-0.33 ± 1.64	-1.23 ± 1.47	0.45 ± 1.37	0.902	0.461	0.372	0.076
BCVA, LogMAR	-0.03 ± 0.04	0.34 ± 0.61	0.14 ± 0.14	0.52 ± 0.78	**0.002**	**0.030**	0.141	0.295
IOP, mmHg	15.35 ± 2.70	16.21 ± 6.28	15.28 ± 3.25	17.00 ± 7.92	0.765	0.973	0.663	0.657
Exophthalmometry, mm	13.83 ± 1.34	15.85 ± 3.53	15.50 ± 1.26	16.14 ± 4.64	0.218	0.071	0.302	0.768
CDR3 clean reads	89,791,638 ± 21,480,234	102,102,541 ± 8,705,207	97,670,963 ± 9,415,117	105,901,035 ± 5,787,281	0.264	0.470	0.108	0.104
D50	6975 ± 2103	1555 ± 824	1040 ± 738	1997 ± 609	**0.002**	**0.001**	**0.003**	**0.038**

Data are presented as Mean ± SD. N, number of people; F, female; M, male; SE, spherical equivalent; BCVA, best corrected visual acuity; IOP, intraocular pressure; *P* value_*1*_, *P* value between HC group and IOI group; *P* value_*2*_, *P* value between HC group and IOI(EF) group; *P* value_*3*_, *P* value between HC and IOI(IN) group; *P* value_*4*_, *P* value between IOI(EF) and IOI(IN) group

### TCR CDR3 sequence and data analysis

The peripheral blood TCR profiles of all subjects were obtained by high-throughput sequencing. After quality control, we obtained the mean TCR-CDR3 clean reads for each sample in the IOI group as 102,102,541 and the mean TCR-CDR3 clean reads for each sample in the HC group as 89,791,638. The minimum value in the IOI group was 82,737,122 and the maximum value in the IOI group was 113,676,620. The minimum value in the HC group was 72,502,404 and the maximum value in the HC group was 132,772,068. Functional analysis of the TCR rearrangement sequences of each sample revealed that more than 85% of the TCR-encoded products with functions were found in each sample. (Supplemental Table S2).

### CDR3 polypeptide chain length distribution characteristics and TRBV and J gene usage

**Fig. 2:**
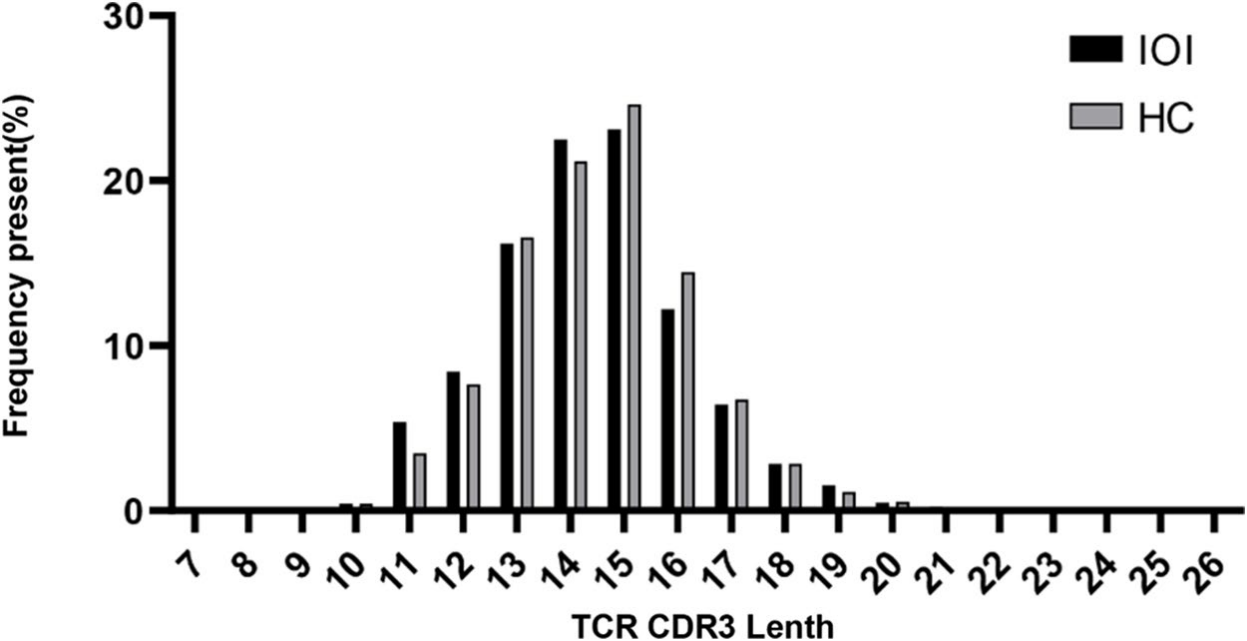
Length distribution of TCR CDR3 in the IOI and the HC groups

The diversity of TCR CDR3 repertoire was correlated with the recombination V, D, and J genes [29]. During recombination, exonuclease sheared the end of the recombinant gene as well as terminal deoxynucleotidyl transferase (TdT) introduced additional nucleotides at the D-J and V-D junctions of the recombinant gene. These processes caused changes in the length of the CDR3 [30, 31]. Therefore, we calculated the cumulative frequencies of V, D, and J structural domains and V-J paired gene use types and CDR3 lengths. We found that the distribution of CDR3 length in the IOI group was similar to that of the HC group, mostly between 13 and 16 nt, with a peak accumulation frequency of 15 nt (Fig. 2).

The length distribution of TCR CDR3 amino acid of the IOI group (n = 13) and HC group (n = 6). The amino acid length showed a peak at 15 nt of both two groups.

**Fig. 3:**
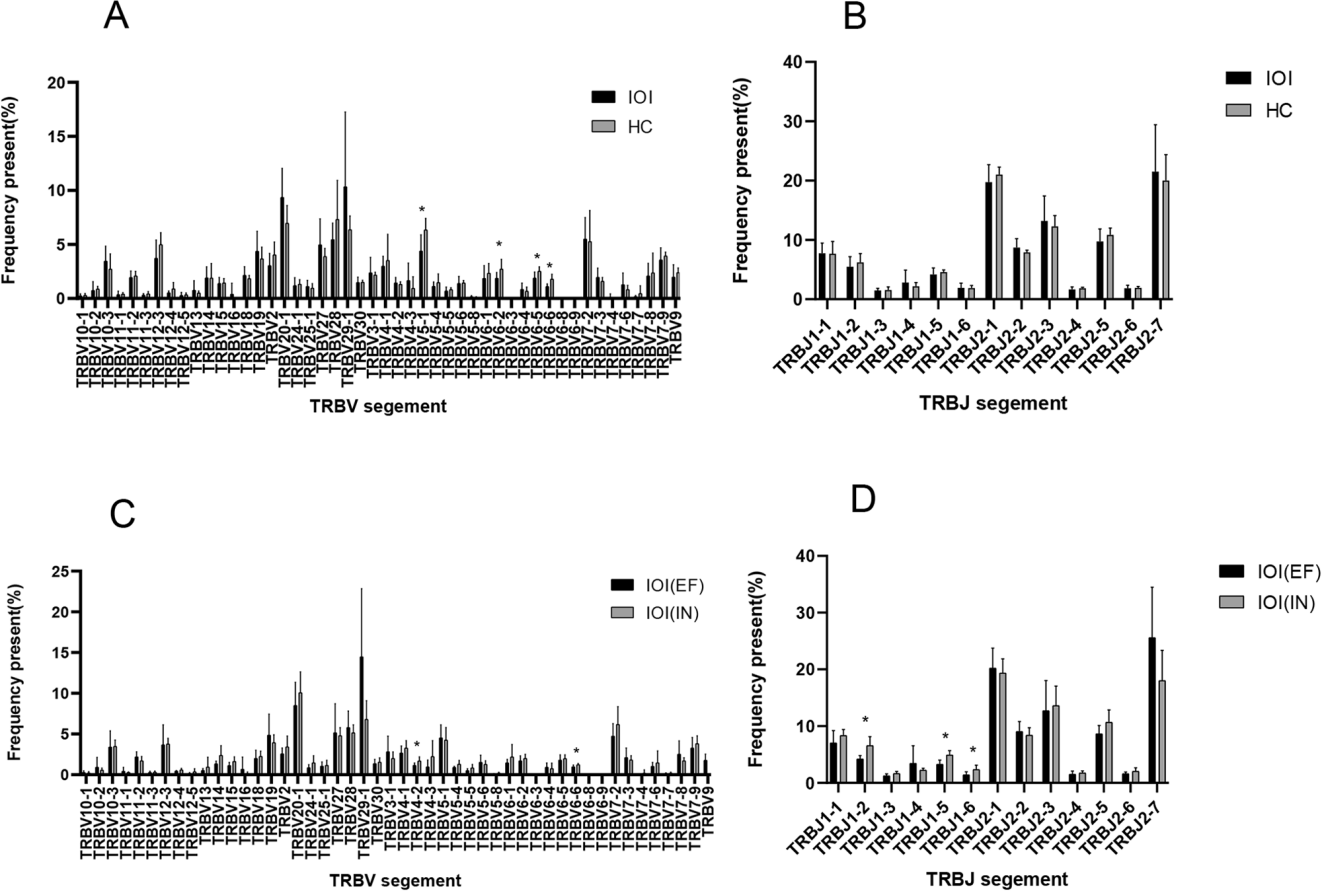
Usage of the V、J segment in the different groups. Frequency distribution of TRBV gene (**A**) and TRBJ gene (**B**) in the IOI group (n = 13) and the HC group (n = 6). Frequency distribution of TRBV gene (**C**) and TRBJ gene (**D**) in the IOI(EF) group (n = 6) and the IOI(IN) group (n = 7)

Although the type of TRBV gene use was essentially the same in the IOI and HC groups, we found significant differences in the frequency of partial gene accumulation in the two groups. Among them, the frequencies of TRBV5-1, TRBV6-2, TRBV6-5, and TRBV6-6 use were significantly lower in the IOI group than in the HC group (P = 0.010, P = 0.019, P = 0.024, P = 0.011) (Fig. 3A).

At the TRBV20-1 and TRBV29-1 sequences, the frequency of use showed a trend of difference that was not significant between the IOI and the HC groups (P = 0.065, P = 0.065) (Fig. 3A). Among the 13 IOI samples before glucocorticoid therapy, the type of V gene use was the same between the IOI(EF) and the IOI(IN) groups, and the frequency of use at TRBV4-2 and TRBV6-6 was significantly higher in the IOI(IN) group than in the IOI(EF) group (P = 0.041, P = 0.032) (Fig. 3C).

In the analysis of the frequency of J gene use, there was no significant difference between the IOI and HC groups (Fig. 3B). However, between the IOI(IN) group and the IOI(EF) group, there were statistically significant differences in the use of sample genes on J1-2, J1-5 and J1-6 (P = 0.005, P = 0.003, P = 0.026) (Fig. 3D).

### TCR CDR3 diversity in the samples

To quantify the TCR diversity in the IOI and HC groups, we used Shannon entropy as well as D50.

**Fig. 4:**
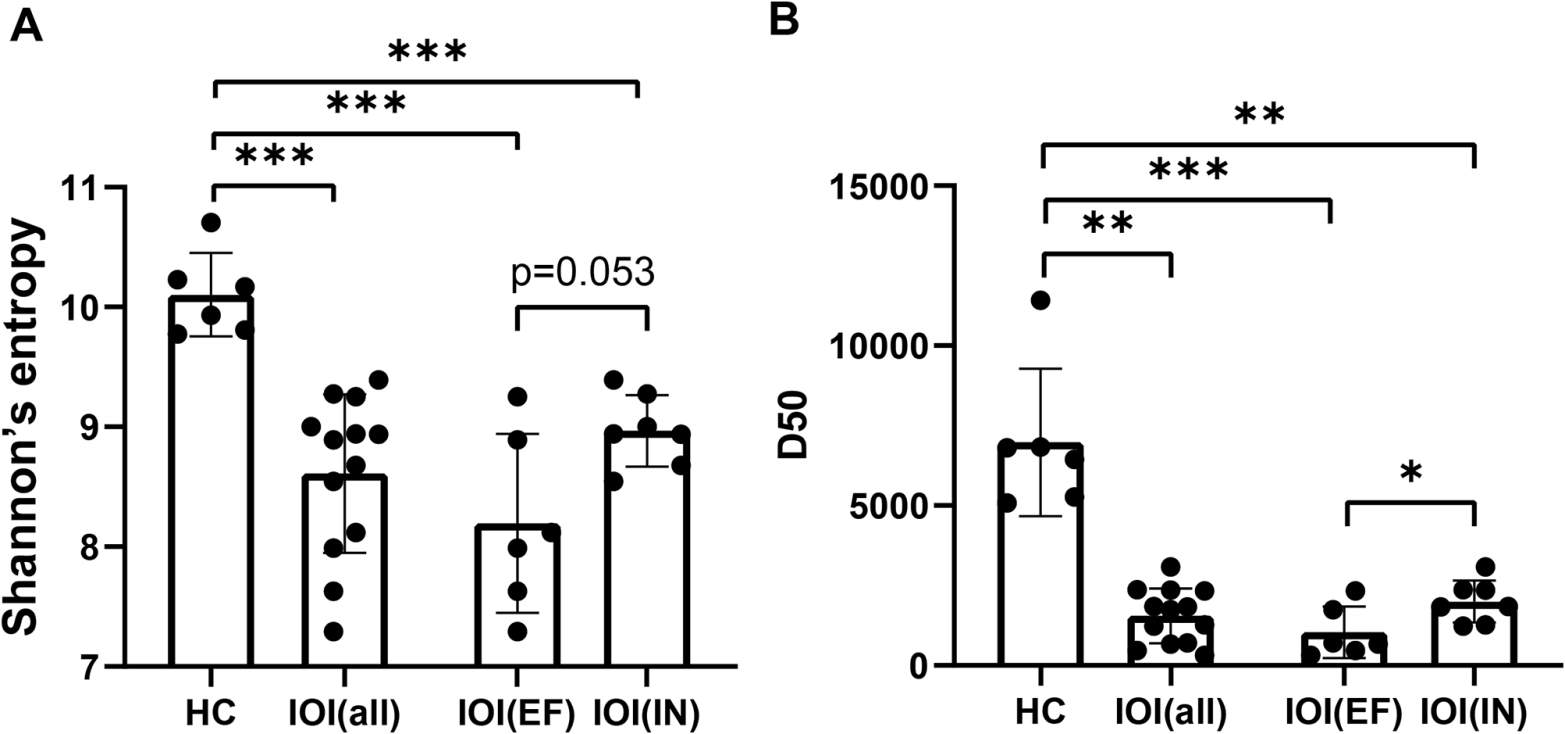
Comparison of diversity among groups (IOI, HC, IOI(EF) and IOI(IN)). Comparison of Shannon entropy (**A**) and D50 (**B**) among groups. The IOI group (n = 13), the HC group (n = 6), the IOI(EF) group (n = 6) and the IOI(IN) group (n = 7)

We analyzed the TCR CDR3 sequences of each group and found that both Shannon entropy and D50 were significantly lower in the IOI group than in the HC group (P < 0.001, P = 0.002). Besides, Shannon entropy and D50 were significantly lower in the IOI(EF) group than in the HC group (P < 0.001, P < 0.001). Shannon entropy and D50 were significantly lower in the IOI(IN) group than in the HC group (P < 0.001, P = 0.003) (Fig. 4A, B). Next, we compared the diversity of the IOI(EF) and the IOI(IN) group before glucocorticoid treatment. Shannon entropy showed a trend of difference that was not significant between the IOI(EF) group and the IOI(IN) group (P = 0.053) (Fig. 4A). Finally, D50 was significantly lower in the IOI(EF) group than in the IOI(IN) group (P = 0.038) (Fig. 4B).

Blood samples were also collected from six IOI patients after glucocorticoid therapy, including four in the IOI(EF) group and two in the IOI(IN) group. By comparing the Shannon entropy before and after treatment, it was found that the diversity of these six samples showed an increasing tendency that was not significant after treatment(P = 0.063). (Supplemental Table S3).

### Patterns of TCR CDR3 sequence sharing among individuals

As mentioned above, the degree of clonal amplification and the frequency of gene accumulation differed between the IOI and HC groups, so it would be interesting to know if there is an overlap between the sequences of different individuals in each group. CDR3 is the most diverse region in the TCR involved in epitope recognition, so our analysis focused on the DNA and amino acid sequences of CDR3.

Notably, we found 133 CDR3 DNA sequences shared among the 13 IOI patients; these corresponded to 131 amino acid sequences, two of which had a frequency of more than 0.01% in each sample. In contrast, there were only three shared nucleotide sequences in the HC group, corresponding to three amino acid sequences. The 133 sequences shared by the IOI group were visible in 0 to 2 samples in the HC group, all with frequencies below 0.01%. Table 2 lists the top 10 nucleotide sequences and their corresponding amino acid sequences in the frequency of the shared sequences among the 13 IOI samples. We show the V-J gene pairs corresponding to these 10 sequences (Supplemental Table S4). The top 10 nucleotide sequences and their corresponding amino acid sequences are visible in some of the HC group samples, but the sequence percentage is extremely low. In addition, 133 CDR3 DNA sequences were shared among all IOI(EF) group samples, and the top 10 sequences with the highest frequencies were all visible in the IOI(IN) group. There were 175 CDR3 DNA sequences shared among all IOI(IN) group samples, and the top 10 sequences with the highest frequencies were all seen in the IOI(EF) group.

**Table 2:** The top 10 frequency sequences in the IOI sample-sharing sequence

nSeqCDR3	aaSeqCDR3
TGCAGCGTTGGGCCCACAAGCTACGAGCAGTACTTC	CSVGPTSYEQYF
TGTGCCAGCAGCCAAGGCGAGGTGCTAGCGGGAGTCCCCGAGCAGTTCTTC	CASSQGEVLAGVPEQFF
TGTGCCAGCAGCCCTAGCGGGAACACTTACGAGCAGTACTTC	CASSPSGNTYEQYF
TGCAGCGTTGAGCGGAGTCATGGGAATGAGCAGTTCTTC	CSVERSHGNEQFF
TGCAGTGCTTCCCCGGGAGTGCCCGGGTCGGCCGAGCAGTACTTC	CSASPGVPGSAEQYF
TGTGCCAGCAGTTTAGATGTGGTGTCCGGGGAGCTGTTTTTT	CASSLDVVSGELFF
TGCAGCGTTGAAAGGCAGCAGAACACTGAAGCTTTCTTT	CSVERQQNTEAFF
TGTGCCAGTAGTACGGGGGGCTTCTACGAGCAGTACTTC	CASSTGGFYEQYF
TGCAGCGTTGGGCCCACAAGCTACGAGCAGTACTTC	CSVGPTSYEQYF
TGTGCCAGCAGCCCGCGCGGGGCGATAGGATCGAACACTGAAGCTTTCTTT	CASSPRGAIGSNTEAFF

nSeqCDR3, the nucleotide sequence of CDR3 sequencing; aaSeqCDR3, amino acid sequence by CDR3 sequencing

## Discussion

In the present study, we used high-throughput sequencing technology to target amplification of the TCR CDR3 region and analyzed the length, distribution of V and J gene use, diversity of CDR3 regions, and information on sharing patterns between individuals in IOI patients compared with healthy subjects, and compared the unique characteristics of the TCR in glucocorticoid-sensitive and glucocorticoid-ineffective patients. To our knowledge, this study is the first TCR sequencing analysis of TCR CDR3 sequences in IOI disease. The main findings of the paper are as follows: (1) The length of TCR CDR3 sequences in the IOI group was approximately similar to that in the HC group. (2) Characteristic TRBV sequences were present in the IOI group, and there were significant differences in TRBV and TRBJ gene expression between the IOI(EF) and the IOI(IN) groups of IOI patients. (3) TCR diversity was suppressed in the IOI group compared with the HC group, and TCR diversity was lower in the IOI(EF) group than in the IOI(IN) group. (4) The shared sequences in the IOI group were much larger than those in the HC group.

The length of the TCR CDR3 region has an important influence on its three-dimensional structure and antigenic specificity [32–34]. Random addition of ligated nucleotides at V-D and D-J, as well as exonuclease nibbling at the ends, can lead to CDR3 length changes [35]. In the present study, we found that the most common nucleic acid sequence length of CDR3 in both the IOI and HC groups was 15 nt. The IOI group was shorter at 16 nt than the HC group and had a similar distribution at the other lengths. Previous studies have shown that the CDR3 gene length in patients with systemic lupus erythematosus (SLE) and severe acne is not significantly different from that of healthy individuals, while the total CDR3 gene length is shorter in patients with psoriasis than in healthy adults [36, 37]. Maryam Yassai et al. found that the short CDR3 structure was associated with thymic CD4 single-positive T cells [38]. Taken together, these findings indicate that the relationship between CDR3 region length and disease development is not yet clear and needs to be further explored in the future.

The complex recombination of VDJC gene fragments and random insertion or deletion of nucleotides in the V-D and D-J junction regions lead to different frequency profiles of TRBV and TRBJ gene use [39]. However, an increasing number of studies have shown the presence of restricted expression or overexpression of TRBV and TRBJ genes in immune-related diseases [36, 37]. In the present study, we analyzed and explored the expression characteristics of the TRBV and TRBJ genes in the TCR CDR3 region of IOI patients. The results showed that the expression of TRBV5-1, TRBV6-2, TRBV6-5, and TRBV6-6 sequences was significantly lower in IOI patients than in healthy control individuals, suggesting that the expression of these TRBV genes is restricted in IOI disease. We predict that the abnormal sequences of the above frequencies in IOI patients may be characteristic sequences of the disease. The frequency of gene use for TRBV4-2, TRBV6-6, TRBJ1-2, TRBJ1-3, and TRBJ2-5 was significantly higher in the IN group than in the IOI(EF) group. These findings propose the abnormal proliferation of T cells encoding these genes in the IOI(IN) group, which may provide a reference for predicting the effect of glucocorticoid therapy based on the differences in gene use characteristics between the IOI(EF) and IOI(IN) groups before treatment.

The TCR CDR3 immune library has been widely used in immune-mediated diseases, and the loss of its diversity is associated with multiple disease states. Previous studies have found reduced diversity of TCR pools in the blood of patients with SLE and psoriasis compared to normal subjects [36, 37]. However, Shao Lei et al. found that blood TCR diversity was elevated in patients with severe acne compared to normal control subjects [40]. In addition, clonal proliferation of T lymphocytes was found to be present in the synovial fluid of lesions in patients with rheumatoid arthritis [41]. Our study showed that the TCR diversity in the IOI group was significantly lower than that in the HC group, suggesting that there is clonal proliferation of certain TCR-specific T cells in IOI disease that disrupts TCR polymorphism. This provides new ideas for the study of the pathogenesis of IOI and new clues for its diagnosis.

Previously, Jiang Yu et al. found that SLE patients treated with glucocorticoids had reduced disease activity and significantly higher blood TCR diversity [42]. A study in microscopic lesion nephrotic syndrome found that many patients had improved TCR diversity at the time of clinical symptom improvement after treatment [43]. In the present study, we found that the TCR diversity in the IOI(EF) group before treatment was lower than that in the IOI(IN) group. This finding may be used to predict the efficacy of glucocorticoid therapy before patient treatment and to avoid unnecessary glucocorticoid side effects. And before making predictions, we need to establish the diversity threshold level first, in addition to sensitivity/specificity and FDR measurements. In the six patients for whom blood samples before and after glucocorticoid treatment were available, there was a nonsignificant trend toward higher diversity, suggesting that the diversity of TCRs in patients with IOI may have been restored to some extent after glucocorticoid treatment, which is consistent with the results reported in the literature above. The current sample size of patients with post-treatment IOI in this study is small, and no significant results have been obtained after subdividing into the IOI(EF) and IOI(IN) groups. We will further expand our sample size in the future to study the changes in TCR diversity before and after treatment in patients with glucocorticoid-sensitive and glucocorticoid-ineffective IOI.

In addition, we analyzed the overlap of the TCR repertoire among individuals to explore the public T-cell response. The analysis revealed a total of 133 nucleotide sequences between all IOI individuals, and the number of amino acids corresponding to the above nucleotide sequences was 131 due to the degeneracy of the codons. Among them, there were 2 sequences whose shared frequency was greater than 0.01%, which might be used as biomarkers for disease risk and diagnosis of IOI. The common nucleotide sequences of IOI group were found in 0 ~ 2 samples of HC group and the frequency was less than 0.01%. In the HC group, however, only 3 nucleotide sequences and amino acid sequences were shared among 6 healthy individuals. This indicates that there are significantly more shared sequences in the IOI group, suggesting the emergence of specific T-cell clonal expansion in IOI. This provides a theoretical basis and molecular target reference for subsequent studies of immune intervention therapy.

In conclusion, this study successfully applied high-throughput immunome library sequencing technology to investigate the TCR CDR3 diversity and immune profile composition characteristics in IOI patients. In addition, we also explored the differences in diversity and gene usage frequency between glucocorticoid-sensitive and glucocorticoid-ineffective IOI patients. The results of this study provide new evidence for further elucidating the role of TCR spectrum in IOI immune response, further exploring the pathogenesis of IOI, and predicting the efficacy of glucocorticoid.

### Electronic supplementary material

Below is the link to the electronic supplementary material.

**Supplementary Material 1**: **Table S1**. Disease activity score of IOI patients before and after treatment. **Table S2**. Basic information of TCR sequencing. **Table S3**. Comparison of diversity before and after glucocorticoid therapy. **Table S4**. V-J gene sequences in the top 10 frequencies of shared sequences of IOI samples

## Data Availability

The available data of this study was deposited in NCBI Sequence Read Archive under accession: PRJNA938605.

## List of abbreviations

IOI: Idiopathic orbital inflammation
TCR: T cell receptor
OIP: orbital inflammatory pseudotumor
HTS: high-throughput sequencing
PBMCs: peripheral blood mononuclear cells
CDR: complementarity-determining region
SE: spherical equivalent
BCVA: best corrected visual acuity
IOP: intraocular pressure
TdT: terminal deoxynucleotidyl transferase
SLE: systemic lupus erythematosus
