# Supplementary material for: Composition and diversity analysis of the TCR CDR3 repertoire in patients with idiopathic orbital inflammation using high-throughput sequencing
Source: BMC Ophthalmol. 2023 Dec 4;23:491. doi: 10.1186/s12886-023-03248-x (PMC10694961; doi:10.1186/s12886-023-03248-x)
Supplement: Supplementary file 1 — Supplementary Material 1: Table S1. Disease activity score of IOI patients before and after treatment. Table S2. Basic information of TCR sequencing. Table S3. Comparison of diversity before and after glucocorticoid therapy. Table S4. V-J gene sequences in the top 10 frequencies of shared sequences of IOI samples [file 12886_2023_3248_MOESM1_ESM.docx]

**Table S1**

Disease activity score of IOI patients before and after treatment

| Sample | Before/After Treatment | Pain | Soft tissue | Proptosis | EOM | Corneal | Sight loss | Total |
| --- | --- | --- | --- | --- | --- | --- | --- | --- |
| IOI01 | Before | 3 | 4 | 0 | 6 | 0 | 6 | 19 |
| (EF) | After | 1 | 2 | 0 | 4 | 0 | 6 | 13 |
| IOI02 | Before | 1 | 4 | 0 | 0 | 0 | 0 | 5 |
| (EF) | After | 0 | 2 | 0 | 0 | 0 | 0 | 2 |
| IOI03 | Before | 4 | 4 | 0 | 6 | 0 | 6 | 20 |
| (EF) | After | 3 | 3 | 0 | 4 | 0 | 6 | 16 |
| IOI04 | Before | 0 | 3 | 4 | 0 | 0 | 0 | 7 |
| (IN) | After | 0 | 3 | 4 | 0 | 0 | 0 | 7 |
| IOI05 | Before | 0 | 4 | 0 | 0 | 0 | 0 | 4 |
| (IN) | After | 0 | 4 | 0 | 0 | 0 | 0 | 4 |
| IOI06 | Before | 0 | 4 | 0 | 6 | 0 | 0 | 10 |
| (IN) | After | 0 | 3 | 0 | 6 | 0 | 0 | 9 |
| IOI07 | Before | 2 | 4 | 0 | 6 | 0 | 8 | 20 |
| (IN) | After | 2 | 4 | 0 | 6 | 0 | 8 | 20 |
| IOI08 | Before | 3 | 3 | 0 | 6 | 0 | 6 | 18 |
| (IN) | After | 3 | 3 | 0 | 6 | 0 | 6 | 18 |
| IOI09 | Before | 1 | 4 | 0 | 6 | 0 | 8 | 19 |
| (IN) | After | 1 | 3 | 0 | 6 | 0 | 8 | 18 |
| IOI10 | Before | 3 | 4 | 0 | 0 | 0 | 0 | 7 |
| (EF) | After | 1 | 2 | 0 | 0 | 0 | 0 | 3 |
| IOI11 | Before | 2 | 4 | 0 | 0 | 0 | 6 | 12 |
| (EF) | After | 1 | 3 | 0 | 0 | 0 | 6 | 10 |
| IOI12 | Before | 1 | 4 | 0 | 6 | 0 | 10 | 21 |
| (IN) | After | 1 | 3 | 0 | 6 | 0 | 10 | 20 |
| IOI13 | Before | 3 | 4 | 0 | 0 | 0 | 0 | 7 |
| (EF) | After | 1 | 2 | 0 | 0 | 0 | 0 | 3 |

EOM: extraocular muscles.

**Table S2**

Basic information of TCR sequencing

| Sample | Total reads(pair) | Aligned Reads (Pair) | Clonotypes | In-frame | Non-function | Out-of-frame |
| --- | --- | --- | --- | --- | --- | --- |
| HC01 | 9013939 | 6996226(77.62%) | 6519774 | 5958407(91.39%) | 284373 | 276994 |
| HC02 | 8974721 | 6746569(75.17%) | 6243654 | 5527470(88.53%) | 476180 | 240004 |
| HC03 | 10491497 | 8331581(79.41%) | 7772084 | 7188389(92.49%) | 249912 | 333783 |
| HC04 | 11465386 | 9342940(81.49%) | 8781523 | 7987624(90.96%) | 387597 | 406302 |
| HC05 | 11619586 | 6894715(59.34%) | 6632055 | 5903945(89.02%) | 344211 | 383899 |
| HC06 | 10115113 | 5436496(53.75%) | 5162286 | 4424422(85.71%) | 417645 | 320219 |
| IOI01 | 3441227 | 2553230(74.20%) | 2303315 | 2120443(92.06%) | 59364 | 123508 |
| IOI02 | 2583203 | 1779222(68.88%) | 1583741 | 1391425(87.86%) | 98275 | 94041 |
| IOI03 | 3125683 | 2161053(69.14%) | 1933927 | 1732846(89.60%) | 76497 | 124584 |
| IOI04 | 4153929 | 2921686(70.34%) | 2618818 | 2359051(90.08%) | 85196 | 174571 |
| IOI05 | 3504659 | 2586190(73.79%) | 2273434 | 2007274(88.29%) | 124648 | 141512 |
| IOI06 | 3944521 | 2867118(72.69%) | 2597383 | 2354424(90.65%) | 98976 | 143983 |
| IOI07 | 2784786 | 2090976(75.09%) | 1824628 | 1639696(89.86%) | 76568 | 108364 |
| IOI08 | 3758126 | 2515677(66.94%) | 2262064 | 2047516(90.52%) | 64558 | 149990 |
| IOI09 | 7992450 | 4097846(51.27%) | 3838770 | 3381260(88.08%) | 232317 | 225193 |
| IOI10 | 6255551 | 3088278(49.37%) | 2777272 | 2457443(88.48%) | 127055 | 192774 |
| IOI11 | 8047271 | 4297762(53.41%) | 4077615 | 3542779(86.88%) | 233532 | 301304 |
| IOI12 | 8160240 | 4222577(51.75%) | 4017150 | 3574946(88.99%) | 162702 | 279502 |
| IOI13 | 9318154 | 5620902(60.32%) | 5348798 | 4980333(93.11%) | 111287 | 257178 |

Total reads, number of reads pairs for comparison after quality control; Aligned Reads Pairs, the number of reads pairs matched to TCR and the proportion of total reads pairs;

In-frame, TCR can encode functional products; Non-function, TCR encodes nonfunctional products; Out-of-frame, TCR cannot be encoded efficiently.

**Table S3**

Comparison of diversity before and after glucocorticoid therapy

| Sample | Before / After Treatment | Shannon entropy | D50 |
| --- | --- | --- | --- |
| IOI01(EF) | Before | 7.987 | 674 |
|  | After | 8.481 | 1195 |
| IOI02(EF) | Before | 7.291 | 471 |
|  | After | 8.456 | 1799 |
| IOI03(EF) | Before | 7.627 | 315 |
|  | After | 8.996 | 1752 |
| IOI10(EF) | Before | 9.252 | 1740 |
|  | After | 10.99 | 13522 |
| IOI07(IN) | Before | 8.679 | 1235 |
|  | After | 10.148 | 6637 |
| IOI12(IN) | Before | 8.544 | 1271 |
|  | After | 8.366 | 703 |

**Table S4**

V-J gene sequences in the top 10 frequencies of shared sequences of IOI samples

| V gene | J gene |
| --- | --- |
| TRBV29-1 | TRBJ2-7 |
| TRBV3-1 | TRBJ2-1 |
| TRBV16 | TRBJ2-7 |
| TRBV29-1 | TRBJ2-1 |
| TRBV20-1 | TRBJ2-7 |
| TRBJ2-2 | TRBJ2-2 |
| TRBV29-1 | TRBJ1-1 |
| TRBV19 | TRBJ2-7 |
| TRBV29-1 | TRBJ2-7 |
| TRBV11-2 | TRBJ1-1 |
